# Supplementary material for: The Role of Adult Vaccines as Part of Antimicrobial Stewardship: A Scoping Review
Source: Antibiotics (Basel). 2023 Sep 10;12(9):1429. doi: 10.3390/antibiotics12091429 (PMC10525636; doi:10.3390/antibiotics12091429)
Supplement: Supplementary file 1 [file antibiotics-12-01429-s001.zip › antibiotics-2572022-supplementary.pdf]

antibiotics

Table S1. Study Protocol Overview

**Scoping review details**	
Scoping review title	The role of vaccines as part of antimicrobial stewardship (AMS): A scoping review
Review objective(s)	1. To examine and map the available evidence supporting the role of vaccines as part of AMS strategies.<br>2. To identify and analyse the gaps in the evidence base when it comes to supporting vaccines as part of AMS strategies.
Review question(s)	1. What types of evidence support the role of vaccines in AMS practices to reduce antimicrobial resistance (AMR)?<br>2. What AMS strategies and/or interventions have been recommended or utilised to support vaccinations?<br>3. What are the current barriers/challenges to vaccinations as part of AMS practices?
**Inclusion/exclusion criteria**	
Participants	Studies (MEDLINE, Embase, Scopus, CINAHL, Cochrane Central Register of Controlled Trials, IPA, and Web of Science) and limited grey literature (.org, .edu, .gov, .int, .au websites) that discuss, examine or explore the use of vaccines in adult patients (≥18 years of age) to reduce AMR as part of AMS strategies. Literature discussing the use of vaccines in adolescent and paediatric patients (<18 years of age) will be excluded.
Concept	The scoping review will consider the concept of immunisation as part of AMS practices in adult individuals or patients with vaccines against both bacterial and viral infections, and what strategies are used to support vaccine uptake. In addition, the current barriers/challenges to vaccination as part of AMS in individuals where Immunisation Schedules do not necessarily mandate vaccine use will be explored.
Context	Studies in English from all geographical locations and healthcare settings will be included.
Types of evidence source	All sources of evidence, including primary studies, reviews, reports, dissertations, and expert opinion articles.

Table S2. Search Strategy Details

MEDLINE

**1**	Antimicrobial Stewardship/
**2**	((Antimicrobial or Antibiotic or Anti-microbial or anti-biotic or anti-infective or antiinfective) and steward*).mp.
**3**	Vaccination/
**4**	vaccines/
**5**	vaccin*.mp.
**6**	Vaccination Coverage/
**7**	Immunization/
**8**	Immunization Programs/
**9**	(Immunis* or Immuniz*).mp.
**10**	Inoculat*.mp.
**11**	booster*.mp.
**12**	1 or 2
**13**	3 or 4 or 5 or 6 or 7 or 8 or 9 or 10 or 11
**14**	12 and 13
**15**	limit 14 to (english and "humans only (removes records about animals)")

Embase

**1**	antimicrobial stewardship/
**2**	((Antimicrobial or Antibiotic or Anti-microbial or anti-biotic or anti-infective or antiinfective) and steward*).mp.
**3**	1 or 2
**4**	immunization/
**5**	vaccination/
**6**	vaccine/
**7**	Vaccin*.mp.
**8**	(Immunis* or Immuniz*).mp.
**9**	Inoculat*.mp.
**10**	booster*.mp.
**11**	4 or 5 or 6 or 7 or 8 or 9 or 10
**12**	3 and 11
**13**	limit 12 to (english and "humans only (removes records about animals)")

Cochrane Central Register of Controlled Trials

**1**	((Antimicrobial or Antibiotic or Anti-microbial or anti-biotic or anti-infective or antiinfective) and steward*).mp.
**2**	Vaccination/
**3**	Vaccines/
**4**	Immunization/ or Immunization Programs/
**5**	vaccin*.mp.
**6**	(Immunis* or Immuniz*).mp.
**7**	Inoculat*.mp.

8 booster*.mp.
9 2 or 3 or 4 or 5 or 6 or 7 or 8
10 1 and 9
11 limit 10 to english language

International Pharmaceutical Abstracts (IPA)

1 ((Antimicrobial or Antibiotic or Anti-microbial or anti-biotic or anti-infective or antiinfective) and steward*).mp.
2 vaccin*.mp.
3 (Immunis* or Immuniz*).mp.
4 Inoculat*.mp.
5 booster*.mp.
6 2 or 3 or 4 or 5
7 1 and 6
8 limit 7 to English

SCOPUS

((TITLE-ABS-KEY (((Antimicrobial or Antibiotic or Anti-microbial or anti-biotic or anti-infective or antiinfective) and steward*)) AND TITLE-ABS-KEY ((Vaccin* or Immunis* or Immuniz* or Inoculat* or booster*))) AND (LIMIT-TO (DOCTYPE,"ar") OR LIMIT-TO (DOCTYPE,"re") OR LIMIT-TO (DOCTYPE,"cp")))

Web of Science

TOPIC: (((Antimicrobial or Antibiotic or Anti-microbial or anti-biotic or anti-infective or antiinfective) and steward*)))
AND TOPIC: (((Vaccin* or Immunis* or Immuniz* or Inoculat* or booster*)))
Refined by: DOCUMENT TYPES: (ARICLE OR REVIEW)

CINAHL

S1 MH "Antimicrobial Stewardship"
S2 TX ((Antimicrobial or Antibiotic or Anti-microbial or anti-biotic or anti-infective or antiinfective) and steward*)
S3 S1 OR S2
S4 MH "Immunization"
S5 MH "Vaccines"
S6 MH "Vaccination Coverage"
S7 MH "Immunization Programs"
S8 TX Vaccin*
S9 TX (Immunis* or Immuniz*)
S10 TX (inoculat*)
S11 TX booster*
S12 S4 OR S5 OR S6 OR S7 OR S8 OR S9 OR S10 OR S11
S13 S3 AND S12

Grey Literature (Google Advanced Search)

site:edu (antimicrobial stewardship AND (immunization OR vaccine OR vaccination OR booster OR inoculation))
site:gov (antimicrobial stewardship AND (immunization OR vaccine OR vaccination OR booster OR inoculation))
site:org (antimicrobial stewardship AND (immunization OR vaccine OR vaccination OR booster OR inoculation))
site:int (antimicrobial stewardship AND (immunization OR vaccine OR vaccination OR booster OR inoculation))
site:au (antimicrobial stewardship AND (immunization OR vaccine OR vaccination OR booster OR inoculation))
